# A gluten-free diet has a different effect on the iron profile of celiac disease and non-celiac gluten-sensitive patients with idiopathic iron deficiency anaemia

**DOI:** 10.22037/ghfbb.v18i2.3139

**Published:** 2025

**Authors:** Mehrdad Haghazali, Mohammad Rostami-Nejad, Abbas Hajfathali, Luca Elli, Mohsen Norouzinia, Hamid Asadzadeh-Aghdaei, Amir Sadeghi, Mahshid Akhavan Rahnama, Azadeh Anbarlou, Haniye Ghasiyari, Hamid Mohaghegh-Shalmani, Mostafa Rezaei-Tavirani, Masiha Amiri, Mohammad Reza Zali

**Affiliations:** 1 *Rajaie Cardiovascular Medical and Research Center, Iran University of Medical Sciences, Tehran, Iran*; 2 *Celiac Disease and Gluten Related Disorders Research Center, Research Institute for Gastroenterology and Liver Diseases, Shahid Beheshti University of Medical Sciences, Tehran, Iran *; 3 *Department of tissue engineering, School of Advanced Technologies in Medicine, Shahid Beheshti University of Medical Sciences, Tehran, Iran*; 4 *Center for Prevention and Diagnosis of Celiac Disease, Fondazione IRCCS Ca' Granda Ospedale Maggiore Policlinico, Milano, Italy*; 5 *Basic and Molecular Epidemiology of Gastrointestinal Disorders Research Center, Research institute for Gastroenterology and Liver Diseases, Shahid Beheshti University of Medical Sciences, Tehran, Iran*; 6 *Lismore Base Hospital North LHD, NSW, Australia*; 7 *Proteomics Research Center, Faculty of Paramedical Sciences, Shahid Beheshti University of Medical Sciences, Tehran, Iran*; 8 *Medical University di Bari Aldo Moro, Bari, Italy*; † These authors contributed equally as a first author.

**Keywords:** Celiac disease, Iron deficiency anaemia, Non-celiac gluten sensitivity, Ferritin, Serum iron

## Abstract

**Aim:**

This study aimed to assess the impact of a six-week gluten-free diet (GFD) on the iron profiles of patients with non-celiac gluten sensitivity (NCGS) and CD.

**Background:**

Iron-deficiency anaemia (IDA) is a significant clinical feature of gluten-related disorders, especially Celiac disease (CD).

**Methods:**

The study included 29 CD patients (mean age 40.28 ± 15.57 years) and 29 NCGS patients (mean age 30.31 ± 7.78 years) presenting with IDA who were enrolled in the study during 2023-2024. Haemoglobin, serum iron, serum ferritin, total iron-binding capacity (TIBC), and transferrin saturation (TSAT) levels were assessed at the beginning and after six weeks of GFD. HLA typing was conducted using the Real-time PCR-based SYBR Green method.

**Results:**

Ferritin levels significantly increased in both CD and NCGS groups after the GFD, from 43.7807 to 50.5279 ng/mL and 23.0862 to 42.9910 ng/mL, respectively. Moreover, serum iron and TSAT levels significantly increased in the NCGS group, from 64.8034 to 81.3466 μg/dL and 19.29 ± 11.70 to 23.99 ± 9.05, respectively (p = 0.003).

**Conclusion:**

The most frequent symptoms in CD and NCGS patients were bloating/bone disease (62.1%) and bone disease (37.9%), respectively. GFD was effective in improving IDA in both CD and NCGS patients. Further research is necessary to assess the therapeutic effect of GFD in patients with gastrointestinal symptoms and IDA.

## Introduction

Celiac disease (CD) is an immune-mediated intestinal disorder triggered by ingesting of gluten-containing foods, including wheat, barley, and rye, in genetically susceptible individuals (1-3). It is characterised by chronic inflammation affecting the mucosa and submucosa of the small intestine, which eventually leads to malabsorption of nutrients such as fat-soluble vitamins, iron, B12, and folic acid (4-6). In addition to CD, non-celiac gluten sensitivity (NCGS) is characterised by both gastrointestinal (GI) and non-GI symptoms related to the ingestion of wheat in individuals without serological or histological evidence of CD (7, 8). NCGS appears to be more common than CD, but available data on NCGS present some uncertainties regarding its diagnosis and management (8-10). 

Historically, CD was thought to cause gastrointestinal symptoms like chronic diarrhoea, abdominal discomfort, bloating, and heartburn (2, 3). However, more than half of the adult population now presents atypical forms dominated by extra-intestinal manifestations, such as iron-deficiency anaemia (IDA), osteoporosis, weight loss, and neurological problems. Therefore, screening for CD in populations presenting these signs and symptoms is essential due to the associated increased mortality (5). Previous studies have demonstrated that IDA is the most common extra-intestinal manifestation in patients with CD (10).

IDA in CD has a multifactorial aetiology, including malabsorption, altered iron uptake in the duodenum (10, 11), and genetic predisposition to IDA (9, 12, 13). The prognosis of IDA in CD is good, with normalization of haemoglobin levels during GFD (14, 15). However, limited data are available on the presence of IDA in NCGS. It remains unclear whether NCGS causes IDA. Patients with gluten sensitivity and severe intestinal inflammation but negative serology have been categorised as having seronegative CD (16). Although a correlation between intestinal damage and the degree of micronutrient deficiency has not been established, the histological aspects of NCGS remain uncertain. 

Additional research is required to determine the percentage of seronegative gluten-sensitive patients with micronutrient deficiencies. Iron deficiency anaemia was also common in our NCGS patients. Therefore, this study aimed to assess the impact of a gluten-free/wheat-free diet on NCGS and CD patients with IDA.

## Methods

### Study population

A cohort of 150 patients with CD was referred to the Research Institute for Gastroenterology and Liver Diseases, Shahid Beheshti University of Medical Sciences (Tehran, Iran), between January 2023 and March 2024. Among them, 29 CD patients (18 females, 62.1%; 11 males, 37.9%) with IDA were enrolled. CD diagnosis was based on the presence of anti-tissue transglutaminase antibodies (anti-tTG) and/or anti-endomysium antibodies (anti-EMA) and mucosal flattening on duodenal histology, in alignment with the most recent international guidelines. SNCG diagnosis was made according to the Salerno criteria (9). Among 200 patients with suggestive symptoms, 29 cases (23 females, 79.3%) with NCGS and IDA were selected.

### Serological analysis

A questionnaire covering demographic data, symptoms, and medication use was completed. A hematologist performed hematological evaluation of anemia to exclude gastrointestinal causes of anaemia. Enrolled patients underwent endoscopy and colonoscopy to rule out other possible causes of IDA, such as small bowel ulcers, chronic infections, inflammatory bowel diseases, drug use, and malignancies. CD and NCGS patients followed a GFD for six weeks, and their iron profiles were evaluated before and after the diet. Patients in both groups did not use iron supplementation during the study period.

Normal haematological values were as follows:

- Iron: Adult males: 59-158 μg/dL; Adult females: 37-145 μg/dL

- Ferritin: Males: 12-300 ng/mL; Females: 12-150 ng/mL

- Haemoglobin (Hb): Males: 13.5-17.5 g/dL; Females: 12.0-15.5 g/dL

- Total iron-binding capacity (TIBC): 240-450 μg/dL

- Transferrin saturation (TSAT): 15-50%

### HLA typing

Genomic DNA was isolated using the salting-out method, and DQ2/DQ8 haplotypes were genotyped using a Real-time PCR System (Applied Biosystems, USA) and SYBR Master Mix (Takara Bio, Inc., Otsu, Japan), following the protocol outlined in a prior study (17). Written informed consent was acquired from all participants. 

### Statistical analysis

The Data are expressed as mean ± standard deviation (SD) or percentages. Statistical analysis was conducted using SPSS software version 20 (IBM Corp., Armonk, NY, USA). Comparisons were made using the Chi-squared test, and a p-value of <0.05 was considered statistically significant.

## Results

### Participant demographics

The study included 29 CD patients (mean age 40.28 ± 15.57 years, range 15-65 years; 62.1% female) and 29 NCGS patients (mean age 30.31 ± 7.78 years, range 18-45 years; 79.3% female). Age and gender showed statistically significant differences between the CD and NCGS groups. The most prevalent symptoms among CD patients were bloating and bone disease (each 62.1%). In NCGS patients, bone disease (37.9%) and bloating (34.5%) were the most frequent symptoms. Diarrhoea and nausea/vomiting were more common in CD patients compared to NCGS patients (p = 0.02 and p = 0.001, respectively). No statistically significant differences were observed between the groups for other symptoms, including weight loss, heartburn, bloating, bone disease, and neurological issues.

At histopathological evaluation, 13.8%, 13.8%, and 72.4% of CD patients were classified as Marsh I, II, and III, respectively. Most NCGS patients were Marsh 0 (89.7%), with only three cases (10.3%) showing Marsh I alterations. Among CD patients, 65.5% carried the HLA DQ2 haplotype, 24.1% had the HLA DQ8 haplotype, 7% carried both, and 3.4% were negative for both. In the NCGS group, 79.7% were negative for DQ2 and DQ8, while 13.7% carried HLA DQ2 and 6.8% carried HLA DQ8.

### Iron profile

In CD patients, the mean serum levels of Hb, ferritin, serum iron, TIBC, and TSAT before GFD were 11.60 ± 1.25 g/dL, 43.78 ± 24.57 ng/mL, 57.25 ± 28.50 μg/dL, 393.75 ± 85.31 μg/dL, and 15.78 ± 9.73%, respectively. After GFD, these levels were 11.90 ± 0.99 g/dL, 50.52 ± 24.24 ng/mL, 61.43 ± 22.96 μg/dL, 394.84 ± 79.69 μg/dL, and 16.0 ± 7.63%, respectively. In NCGS patients, the mean serum levels of Hb, ferritin, serum iron, TIBC, and TSAT before GFD were 12.15 ± 1.35 g/dL, 23.08 ± 13.45 ng/mL, 64.80 ± 37.14 μg/dL, 352.48 ± 56.98 μg/dL, and 19.29 ± 11.70%, respectively. After GFD, these levels were 12.46 ± 0.90 g/dL, 42.99 ± 22.70 ng/mL, 81.34 ± 28.37 μg/dL, 348.93 ± 56.89 μg/dL, and 23.99 ± 9.05%, respectively. Only ferritin levels showed a significant increase in CD patients after six weeks of GFD (p = 0.03). In NCGS patients, ferritin (p = 0.001), serum iron (p = 0.003), and TSAT (p = 0.003) levels significantly increased after GFD.

## Discussion

Approximately 20% of CD patients present with IDA. This prospective study confirms CD is a common cause of IDA in adults. Previous data have shown that anaemia generally resolves with strict adherence to a GFD in adult CD patients with IDA (15, 16). The normalisation of duodenal histological lesions during GFD appears to enhance dietary iron absorption, leading to the improvement of anaemia in patients with CD (4, 7, 12, 15, 16).

This study assessed the impact of GFD on the iron profile of NCGS and CD patients with IDA after six weeks. The results showed significant improvements in serum iron, TIBC, and ferritin levels in NCGS patients after GFD. In CD patients, only ferritin levels exhibited a significant rise after six weeks of GFD. The prevalence of CD in IDA patients varies across studies, ranging from 0% to 14.6% (18-21). Limited literature is available on NCGS, with only one case study reporting the reversal of symptoms and normalization of iron profile in a 56-year-old female with IBS and IDA after three months of GFD (22).

The recovery of IDA and increased haemoglobin concentrations may be associated with the progressive restoration of intestinal mucosal integrity after eliminating gluten from the diet (23). Due to intestinal mucosal damage and villous atrophy, oral iron supplementation may not be effectively absorbed in CD patients (24). Therefore, oral iron supplementation therapy for patients with CD is recommended after six months of GFD (25). Annibale et al. (15) reported that 77.8% of CD patients recovered from IDA after six months of GFD, and 94.4% recovered after 12-24 months. Their findings support that anaemia improvement begins after six months of GFD due to the normalization of intestinal mucosal histology. Similarly, our results show that ferritin levels significantly increased in CD and NCGS patients after six weeks of GFD.

## Conclusion

In conclusion, we found that iron plays a crucial role in IDA, the most essential extra-intestinal manifestation of CD that damages the duodenal mucosal surface, leading to impaired absorption. The current opinion is that GFD presents the only nutritional therapy for CD and IDA malabsorption in patients with CD, which easily be improved with a GFD following the restoration of standard histological structure in the intestinal mucosa. Also, in routine evaluation, medical doctors may fail to consider NCGS as a cause of IDA in patients with nonspecific symptoms and negative for CD. Therefore, although gluten- nutrition was associated with improved symptoms of NCGS in IDA, patients, further research is needed to investigate NCGS cases.
